# Supplementary material for: The Deubiquitinase USP39 Promotes Esophageal Squamous Cell Carcinoma Malignancy as a Splicing Factor
Source: Genes (Basel). 2022 May 3;13(5):819. doi: 10.3390/genes13050819 (PMC9141838; doi:10.3390/genes13050819)
Supplement: Supplementary file 1 [file genes-13-00819-s001.zip › Supplementary Figures S1--S3.pdf]

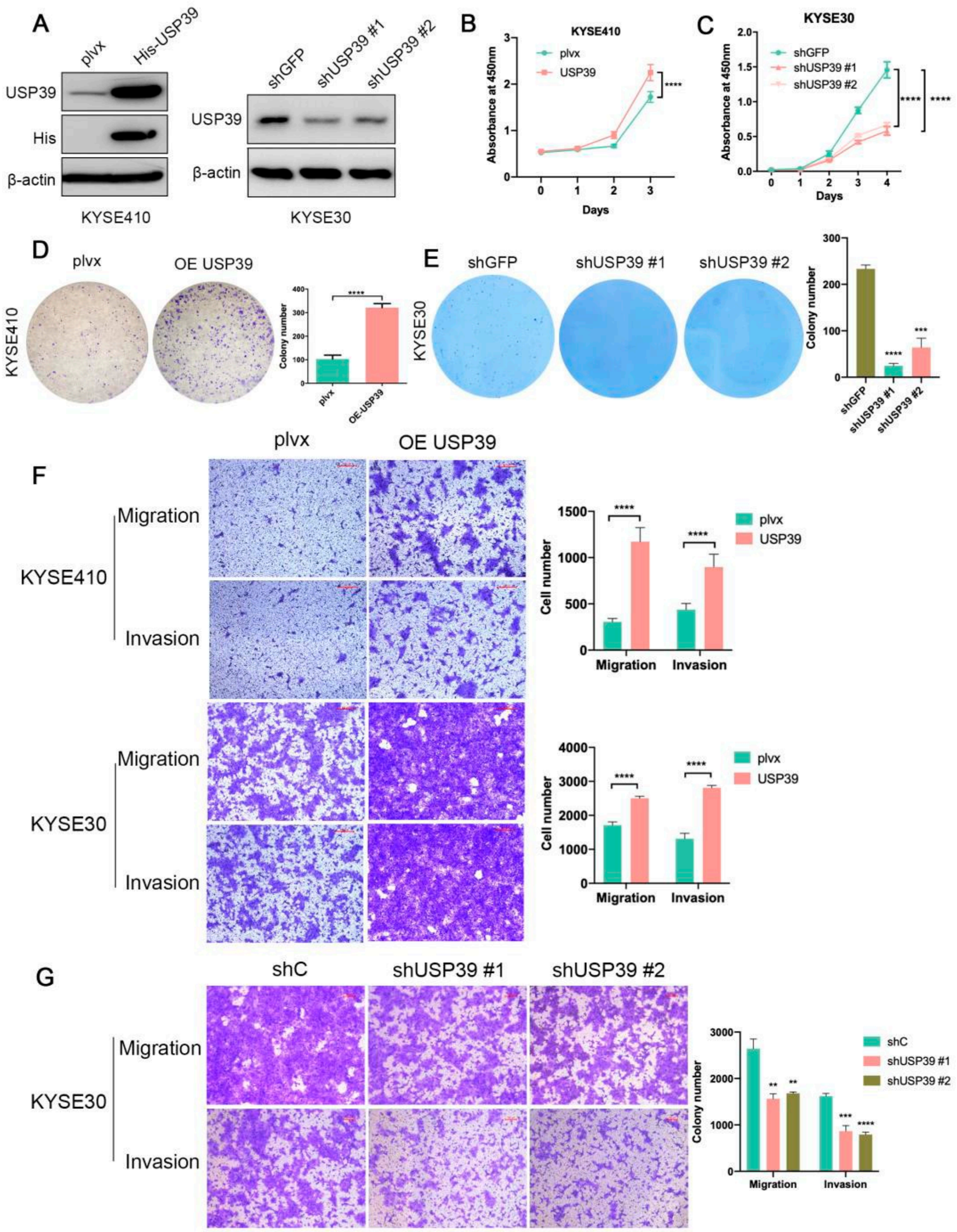

Supplementary Figure S1. USP39 promotes ESCC malignancy. A. Overexpression (Left panel) and knockdown (Right panel) efficiency of USP39 in ESCC cells was determined by Western Blot. **B.** Cell proliferation detected by CCK-8 assay after USP39 overexpression in KYSE410 cells. **C.** Cell proliferation detected by CCK-8 assay after USP39 knockdown in KYSE30 cells. **D.** Cell proliferation detected by colony formation assays after USP39 overexpression in KYSE410. **E.** Cell proliferation

detected by colony formation assays after USP39 knockdown in KYSE30. **F.** Migration and invasion assays were performed using USP39 stable overexpression KYSE410 and KYSE30 cells and corresponding control cells. **G.** Migration and invasion assays were performed using USP39 stable knockdown KYSE30 cells and corresponding control cells. Data in B-G represent mean ± SD. Data were analyzed by two-way ANOVA (B, C) with Bonferroni correction or unpaired two-tailed Student's t-test (D-G). ns = no significant, * $p < 0.05$, ** $p < 0.01$, *** $p < 0.001$, **** $p < 0.0001$.

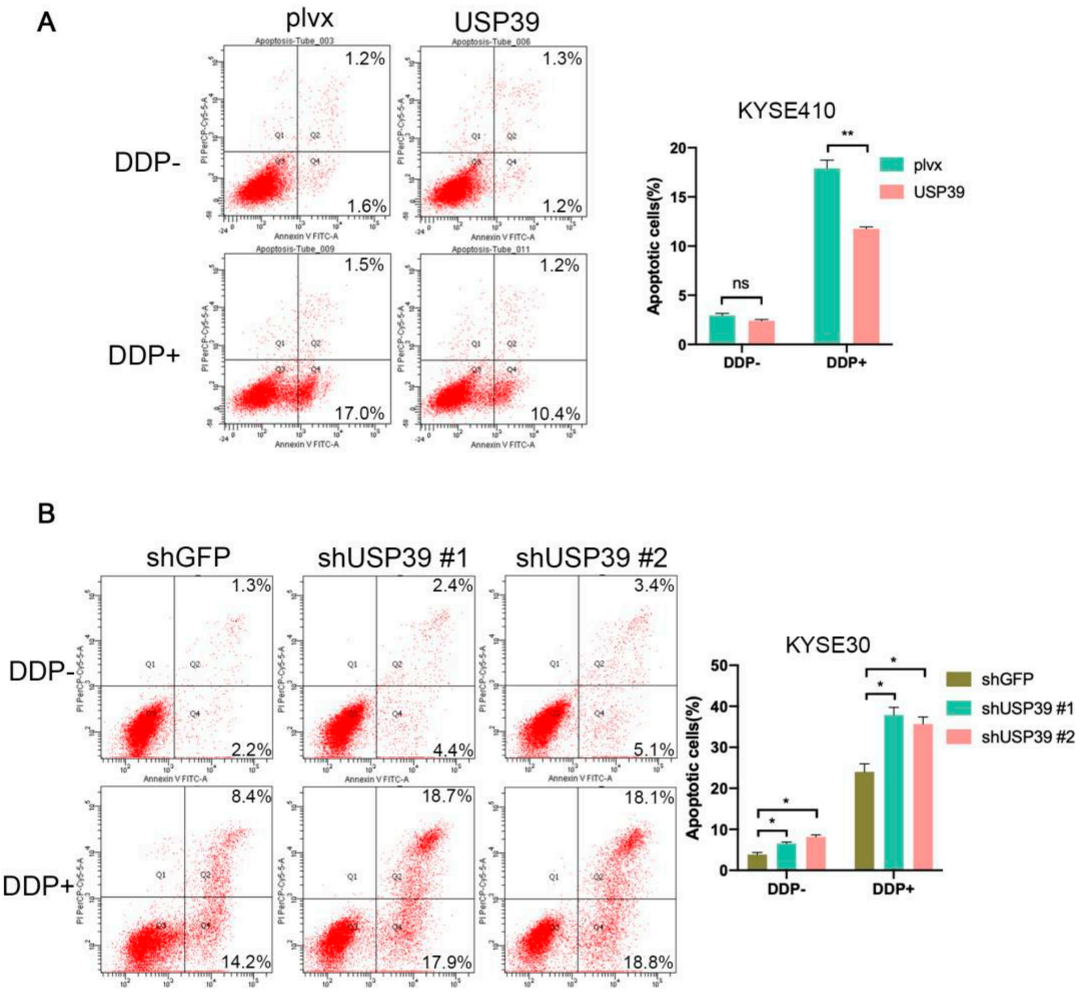

Supplementary Figure S2. USP39 promotes chemoresistance in ESCC cells. A. Cell apoptosis detected by Annexin V-PI assay in USP39-overexpressed KYSE410 and control cells with or without DDP (10μg/ml) treatment. **B.** Cell apoptosis detected by Annexin V-PI assay in USP39-knockdown KYSE30 and control cells with or without DDP (14μg/ml) treatment. Data in A, B represent mean ± SD and were analyzed by unpaired two-tailed Student's t-test. ns = no significant, * $p < 0.05$, ** $p < 0.01$, *** $p < 0.001$, **** $p < 0.0001$.

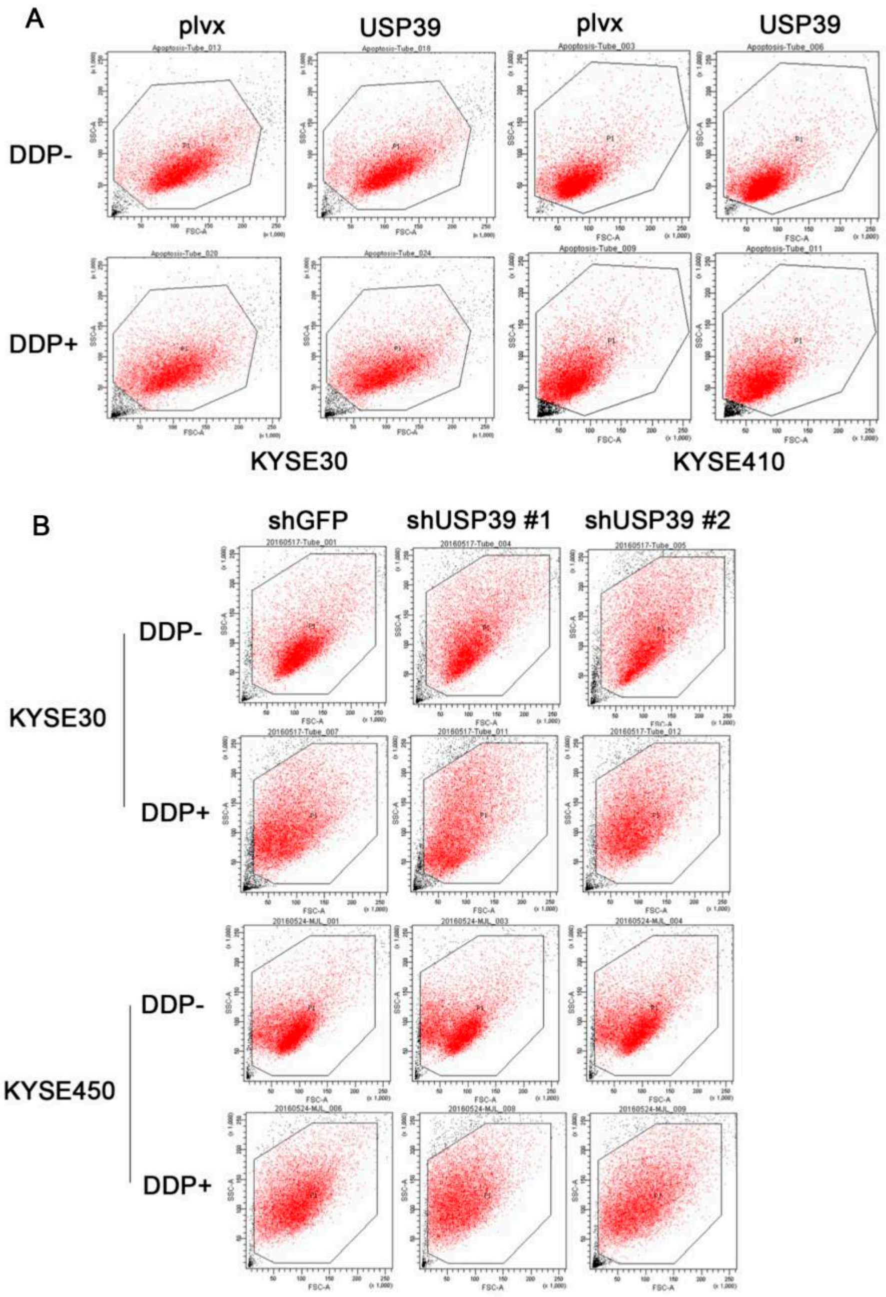

Supplementary Figure S3. The gating strategy used for apoptosis analysis. A. The gating strategy used for apoptosis analysis of KYSE30 and KYSE410 overexpressed USP39 with DDP treatment. **B.** The gating strategy used for apoptosis analysis of KYSE30 and KYSE450 knockdown USP39 with DDP treatment.
